# Insomnia and intellect mask the positive link between schizotypal traits and creativity

**DOI:** 10.7717/peerj.5615

**Published:** 2018-09-17

**Authors:** Bertalan Polner, Péter Simor, Szabolcs Kéri

**Affiliations:** 1 Department of Cognitive Science, Budapest University of Technology and Economics, Budapest, Hungary; 2 Institute of Psychology, Eötvös Loránd University, Budapest, Hungary; 3 Institute of Behavioural Sciences, Semmelweis University, Budapest, Hungary; 4 Nyírő Gyula Hospital, National Institute of Psychiatry and Addictions, Budapest, Hungary; 5 Department of Physiology, University of Szeged, Szeged, Hungary

**Keywords:** Schizotypy, Insomnia, Mental health, Creativity, Resilience, Schizophrenia, Psychosis, Sleep disturbances, Intellect

## Abstract

**Background:**

Schizotypy is a set of personality traits that resemble the signs and symptoms of schizophrenia in the general population, and it is associated with various subclinical mental health problems, including sleep disturbances. Additionally, dimensions of schizotypy show specific but weak associations with creativity. Given that creativity demands cognitive control and mental health, and that sleep disturbances negatively impact cognitive control, we predicted that positive, impulsive and disorganised schizotypy will demonstrate stronger associations with indicators of creativity, if the effect of mental health, insomnia, and intellect are statistically controlled.

**Methods:**

University students (*N* = 182) took part in the study. Schizotypy was assessed with the shortened Oxford-Liverpool Inventory of Feelings and Experiences (sO-LIFE). Creative achievements were measured with the Creative Achievement Questionnaire (CAQ), divergent thinking was assessed with the ‘Just suppose’ task, and remote association problem solving was tested with Compound Remote Associate (CRA) problems. Mental health was assessed with the 12-item version of the General Health Questionnaire (GHQ-12), and insomnia was examined with the Athens Insomnia Scale (AIS). Verbal short term memory was measured with the forward digit span task, and intellect was assessed with the Rational-Experiential Inventory (REI). Multiple linear regressions were performed to examine the relationship between creativity and schizotypy. Indicators of creativity were the dependent variables. In the first block, dimensions of schizotypy, age, gender and smoking were entered, and in the second block, the models were extended with mental health, insomnia, verbal short term memory, and intellect.

**Results:**

Positive schizotypy positively predicted real-life creative achievements, independently from the positive effect of intellect. Follow-up analyses revealed that positive schizotypy predicted creative achievements in art, while higher disorganised schizotypy was associated with creative achievements in science (when intellect was controlled for). Furthermore, disorganised schizotypy positively predicted remote association problem solving performance, if insomnia and verbal short term memory were statistically controlled. No dimension of schizotypy was significantly associated with divergent thinking.

**Discussion:**

In line with previous findings, positive schizotypy predicted real-life creative achievements. The positive effects of disorganised schizotypy might be explained in terms of the simultaneous involvement of enhanced semantic priming and cognitive control in problem solving. We speculate that the lack of associations between divergent thinking and schizotypy might be related to instruction effects. Our study underscores the relevance of sleep impairment to the psychosis-spectrum, and refines our knowledge about the adaptive aspects of schizotypy in the general population.

## Introduction

Schizotypy is often regarded as a set of quasi-pathological personality traits with milder forms of schizophrenia-like symptoms (e.g., odd beliefs, suspiciousness, perceptual anomalies, loosened associations, and peculiar speech) (e.g.,  Kwapil et al., 2013; Nelson et al., 2013; Ettinger et al., 2014). Schizotypy (abbreviating schizophrenic phenotype) was first conceptualised by Rado (1953) as a genetically predisposed personality organization that is present in a subset of the population who are at heightened risk for schizohprenia. Rado speculated that adaptive processes (e.g., intelligence and social support) may help schizotypes compensate, and that psychotic phenomena (e.g., paranoia, hallucinations, and thought disorder) emerge with decompensation. Drawing upon Rado’s ideas, Meehl (1962) hypothesised that the genetic predisposition causes *schizotaxia*, a neural integrative deficiency, which is not only associated with cognitive slippage but also forms the basis for developing schizotypal personality. Importantly, Meehl highlighted that favourable early social experiences and adaptive personality traits (e.g., resilience and reduced trait anxiety) will prevent schizotypes from developing schizophrenia. Contrasting these clinically oriented conceptualisations, Claridge (1994) made a case for a fully dimensional perspective and regarded schizotypy as a trait continuously distributed in the general population that not only indicates liability to schizophrenia but also covers potentially adaptive variation in normal personality; Claridge also theorised that conversion to schizophrenia is a function of other inherited traits, dysfunctions, and environmental effects (also see Grant, Green & Mason, 2018; Lenzenweger, 2018 for recent overviews of schizotypy concepts). Although the relationship between the genetic backgrounds of schizotypy and schizophrenia is still controversial, it has been argued that studying schizotypy in the non-clinical population can provide valuable information about resilience to schizophrenia and psychotic disorders (Barrantes-Vidal, Grant & Kwapil, 2015).

Relatedly, Mohr & Claridge (2015) suggested that positive schizotypy (magical ideation and perceptual anomalies) may be linked to spiritual wellbeing, flexible and creative thinking, and openness to experience. In contrast, negative schizotypy (anhedonia and social isolation) and disorganized schizotypy (loosened thinking and speech, and inadequate emotional reactions) are associated with impaired adaptation to environmental challenges and worse quality of life. Notably, in a meta-analysis of 45 studies, the sole predictor of creativity was the type of schizotypy (Acar & Sen, 2013). Results indicated that the positive-impulsive and unspecified dimensions predicted better creativity (*r* = 0.14), meanwhile the negative-disorganized dimension was associated with worse creativity (*r* = −0.09). Interestingly, larger effect sizes were found for artistic (*r* = 0.21) as compared to general creativity (*r* = 0.06), and for studies conducted with eminent subjects (*r* = 0.20), relative to those that involved non-eminent participants (*r* = 0.06). However, the larger effects should be considered exploratory, as they were based on few studies. No significant moderation effect was found for gender, measurement of creativity (performance vs. self-report, verbal vs. figural, fluency vs. originality), or the measure of schizotypy (comprehensive inventory vs. specific scale). Thus, the meta-analysis revealed that schizotypy dimensions showed specific and weak associations with various indicators of creativity; at the same time, it was limited in that (1) it did not consider whether the schizotypy measure was personality- vs. psychopathology-orientated (Grant, Green & Mason, 2018; Oezgen & Grant, 2018), and failed to make a distinction either between (2) divergent thinking and remote association problem solving abilities (studies have shown no or weak positive correlation between them: (Taft & Rossiter, 1966; Akbari Chermahini, Hickendorff & Hommel, 2012; Lee & Therriault, 2013; Lee, Huggins & Therriault, 2014) or (3) between disorganised and negative schizotypy. The latter might be problematic in that the content of disorganisation scales vary across instruments (Oezgen & Grant, 2018) and that depending on the instrument used, positive schizotypy may show stronger coupling with disorganisation than with negative schizotypy (e.g.,  Venables & Rector, 2000; Mason & Claridge, 2006; Oezgen & Grant, 2018).

Indeed, it could be crucial to differentiate between disorganised vs. negative, and positive vs. impulsive-asocial schizotypy in elucidating their relationship with creativity. For instance, when participants had to construct scenes from randomly chosen shapes, positive schizotypy predicted greater productivity but reduced originality, while disorganised schizotypy predicted greater originality (LeBoutillier, Barry & Westley, 2014). Relatedly, a positive correlation between disorganised schizotypy and remote association problem solving has been documented in a study (Gibson, Folley & Park, 2009). Disorganisation and other dimensions of schizotypy appear to be elevated among artists: higher levels of positive, disorganised and impulsive-asocial schizotypy have been reported in poets (Mason, Mort & Woo, 2015), actors, and comedians (the latter group has also shown elevated negative schizotypy) (Ando, Claridge & Clark, 2014). Another study has reported that relative to non-artists, a group of visual artists not only produced more unique ideas on verbal divergent thinking tasks, but also demonstrated elevated positive, disorganised and impulsive-asocial schizotypy (Burch et al., 2006). The latter finding could indicate that pursuing visual arts is associated with a tendency for taboo breaking, which also could explain artists’ more unique responses on divergent thinking tasks. In line with the above findings, a meta-analysis has shown a positive relationship between psychoticism (a construct involving impulsivity and asocial behaviours) and indicators of creativity (*r* = 0.16), and follow-up analyses revealed a large effect size (*r* = 0.50) if psychoticism was measured with the Eysenck Personality Questionnaire (Eysenck & Eysenck, 1975) and creativity was indicated by the uniqueness of ideas. Although psychoticism was conceptualised by Eysenck as a ‘dispositional trait underlying susceptibility to the development of psychotic symptoms’ (1993, p. 155), it has been argued that at the conceptual level, psychoticism might be closer to psychopathy than to schizotypy (see Grant, Green & Mason, 2018). Relatedly, in more recent studies, psychoticism and hypomania, but not overall schizotypy predicted real-life creative achievements and rated creativity of divergent thinking (Zabelina, Condon & Beeman, 2014); whereas in another study, the impulsive-asocial dimension was the only aspect of schizotypy that predicted insight problem solving performance (Stanciu & Papasteri, 2018), an ability conceptually overlapping with remote association problem solving (Bowden et al., 2005). To the contrary, in a study by Webb et al. (2017), dimensions of schizotypy did not show significant associations neither with performance on insight and remote association problem solving tasks nor with convergent thinking. Although positive relationships emerged between positive schizotypy and aspects of divergent thinking, these associations were not significant in a replication sample.

Creativity is not a unitary construct: successful achievements require a special constellation of creative potential (e.g., divergent thinking and remote association problem solving), expertise, relevant personality traits (e.g., openness to experience and positive schizotypy), and behavioural execution (Amabile, 1983). One can define divergent thinking as a potential to generate multiple original ideas spontaneously and fluently in relation to a given problem, often using distant semantic associations. Benedek et al. (2014) showed that certain components of cognitive control predicted divergent thinking, that is, how people could update and inhibit representations in their active short-term memory. Updating was closely related to fluid intelligence, and, together with the personality trait openness, significantly explained the co-variance between divergent thinking and intelligence. In another study, openness, and fluency and originality of divergent thinking predicted engagement in creative activities, which, in turn, was associated with creative achievements (Jauk, Benedek & Neubauer, 2013). Curiously, intelligence not only predicted creative achievements, but also moderated the relationship between creative activities and achievements.

Beyond intelligence and cognitive control, reduced attentional filtering may also contribute to creativity (Fink et al., 2014a). Latent inhibition refers to the inhibitory effect of non-reinforced stimulus exposure on subsequent performance on tasks involving the pre-exposed stimulus (Lubow & Moore, 1959). Latent inhibition is crucial to selective attention in that it allows filtering stimuli that were previously experienced as irrelevant (Lubow, 2005). Carson, Peterson & Higgins (2003) found that the combination of high intelligence and reduced latent inhibition was associated with outstanding creative achievements, while Kéri (2011) reported that reduced latent inhibition, higher intelligence and a larger primary social network were independent predictors of creative accomplishments. In light of the above, certain cognitive correlates of schizotypy could be expected to show opposing associations with creativity. Indeed, positive schizotypy and divergent thinking were both correlated with less typical free associations, while top-down auditory inhibition was negatively related to positive schizotypy but positively to divergent thinking (Rominger et al., 2017)*.* Reduced latent inhibition might be a common neurocognitive characteristic underlying the link between psychosis proneness and creativity (Eysenck, 1993; Carson, Peterson & Higgins, 2003; Kéri, 2011; Fink et al., 2012). Moreover, divergent thinking and schizotypy appear to share neural substrates: original ideas and overall schizotypy were both related to reduced deactivation in the right precuneus during divergent thinking, which may mirror task-irrelevant mental processes related to broad memory search (Fink et al., 2014b). On the other hand, poor executive functions may hinder creative output in highly schizotypal individuals: according to a meta-analysis, both positive and negative schizotypy were associated with impaired shifting, and negative schizotypy was also linked to weaker updating performance (Steffens et al., 2018).

Although positive schizotypal traits may have certain benefits in the non-clinical population (Mohr & Claridge, 2015), schizotypy is associated with subclinical mental-health problems and subtle cognitive impairments (Nelson et al., 2013; Ettinger et al., 2014; Steffens et al., 2018) that may interfere with creativity. For instance, among students, positive and negative schizotypy were both positively correlated with depression and anxiety (Lewandowski et al., 2006). Moreover, positive and negative schizotypy were both found to predict lower global functioning, schizotypal and paranoid symptoms; positive schizotypy specifically predicted the use of alcohol and drugs and related impairment, plus affective disorders and psychiatric treatment, whereas negative schizotypy was specifically associated with schizoid symptoms, and the lack of intimate relationships (Kwapil, Barrantes-Vidal & Silvia, 2008). Schizotypal traits possess predictive validity (see Debbané et al., 2015 for a review): higher positive and negative dimensions at baseline predicted psychotic-like, schizotypal and paranoid symptoms and reduced functioning 10 years later (Kwapil et al., 2013). What is more, positive schizotypy was specifically associated with affective disorders, substance abuse and receiving mental health treatment, while negative schizotypy was uniquely linked to subsequent schizoid symptoms and reduced social functioning. Additionally, schizotypy has also been linked to worse quality of life, and the most remarkable associations emerged with the negative dimension (Cohen & Davis, 2009). Interestingly, the association between paranormal experiences and mental well-being might be moderated by cognitive disorganisation: in a study, less disorganised participants were more likely to have a belief framework, and for them, positive schizotypy was related to more pleasant paranormal experiences, while for more disorganised participants, negative schizotypy predicted less pleasant paranormal experiences (Schofield & Claridge, 2007).

Particular emphasis should be placed on sleep disorders, which have a major adverse effect on cognitive control (Krause et al., 2017). Patients with schizophrenia suffer from various forms of sleep disorders (insomnia, obstructive sleep apnea, restless leg syndrome, and periodic limb movement disorder) (Kaskie, Graziano & Ferrarelli, 2017), and, by taking into account the theory of schizophrenia-schizotypy continuum, it is likely that similar sleep problems could be detected in non-clinical individuals with schizotypal traits. This hypothesis has a neurobiological basis. Patients with schizophrenia show a decrease in sleep spindles, which are 12–15 Hz phasic oscillations during non-rapid eye movement (NREM) sleep (Ferrarelli et al., 2007). Reduced sleep spindle activity and signs of attenuated slow wave (1–4 Hz) activity were evidenced among first-degree relatives, compared to healthy controls (D’Agostino et al., 2018). The reduction of sleep spindles, reflecting impaired thalamo-cortical interactions, is related to memory disorders and decreased cognitive control (Keshavan et al., 2011; Wamsley et al., 2012; Ujma et al., 2014; Schilling et al., 2017). In addition, slow oscillatory activity reflecting the synchronized activity of a large number of cortical neurons (Achermann et al., 1993; Steriade, McCormick & Sejnowski, 1993) during NREM sleep seems to be critical for higher-level cognitive functions (Walker, 2009; Mander et al., 2011). Strikingly, Lustenberger et al. (2015) found that sleep spindle density was inversely correlated with magical ideation, a positive schizotypal trait, in healthy young male subjects. Therefore, we can assume that sleep disorder has an adverse effect on creative thinking skills, and that due to insomnia and other subclinical mental health problems, the relationship between positive schizotypy and creativity will be weaker.

The present study set out to test several predictions. We expected that indicators of creativity (divergent thinking, remote association problem solving and real-life creative achievements) will be positively predicted by positive and impulsive schizotypy, and negatively by negative schizotypy (Acar & Sen, 2013). Although the theoretical relevance and the measurement of the impulsive-asocial dimension of schizotypy are somewhat controversial (e.g.,  Lin et al., 2013; Fonseca-Pedrero et al., 2015), we nevertheless decided to include impulsive-asocial schizotypy in the analyses as several studies have underscored its connection with creativity (e.g.,  Burch et al., 2006; Acar & Runco, 2012; Acar & Sen, 2013; Stanciu & Papasteri, 2018). Based on studies that distinguished between negative and disorganised schizotypy and found the latter to be positively linked to creativity (Gibson, Folley & Park, 2009; Ando, Claridge & Clark, 2014; LeBoutillier, Barry & Westley, 2014; Mason, Mort & Woo, 2015), we expected that disorganised schizotypy will positively predict creative thinking skills and creative accomplishments. We expected to find stronger associations between positive and impulsive schizotypy and creativity in the domain of art (Acar & Sen, 2013). In addition, as the confounding effects of mental health complaints (Kwapil et al., 2013) and cognitive impairment (Steffens et al., 2018) might have introduced heterogeneity into the literature on creativity in schizotypy*,* we predicted that the positive effects of positive, impulsive and disorganised schizotypy will be more pronounced when their adverse correlates, such as impaired general mental health, insomnia and reduced cognitive capacities, are statistically controlled.

## Materials & Methods

### Sample

Participants were recruited from courses at two major universities in Budapest, and were offered extra points at the courses in exchange for participation. Participants were informed that the aim of the study was to explore the associations between problem solving, creativity and personality. The study was approved by the Ethical Review Committee for Research in Psychology (EPKEB, approval number: 2014/3), and participants provided written informed consent. In total, 182 Hungarian speaking participants completed the laboratory testing session and filled out all questionnaires online (mean (sd) of age = 22.2 (4.2); 58% female). A subset of this sample has been analysed in a previous paper (Simor & Polner, 2017).

### Instruments

#### Creative achievements and creative thinking skills

Lifetime creative achievements were assessed with the Creative Achievements Questionnaire (CAQ; Carson, Peterson & Higgins, 2005; Hungarian adaptation: Kéri, 2011). The CAQ inquires about recognition of creativity in 10 real-life domains (visual arts, music, dance, architectural design, creative writing, humour, inventions, scientific discovery, theatre and film, and culinary arts). In general, accomplishments at higher levels (e.g., national recognition) receive higher scores. The total CAQ score is the sum of the domain scores. We also calculated subscores for accomplishments in art (visual arts, music, dance, creative writing, humour, theatre and film) and science (inventions and scientific discovery) (following Carson, Peterson & Higgins, 2005; Kaufman et al., 2016).

Verbal divergent thinking was measured with the ‘Just suppose’ test (Torrance, 1974). In this task, participants are asked to list their ideas about what would happen if a fantastic scenario happened. The test was introduced as a ‘game-like’ task and the experimenter aimed to create a relaxed atmosphere. Participants were provided three minutes and were instructed to write down as many original and creative ideas as they could. Two forms of the test were applied in the study (1st form: ‘Just suppose people could get anywhere immediately by simply closing and opening their eyes, or by pulling their earlobes.’; 2nd form: ‘Just suppose clouds had strings attached to them which hang down to earth.’). The creativity of each idea was evaluated using the subjective scoring method proposed by Silvia and co-workers (see Silvia et al., 2008 for details). We asked three university students to rate the ideas, who indicated their judgements using a program written in PsychoPy2 (Peirce, 2007). First, raters were shown a detailed definition of creativity. Then, a randomly selected 10–10% of the ideas collected on both forms of the ‘Just suppose’ test were presented, in order to provide raters an overview of typical ideas. Then, raters were shown all the ideas collected on both forms, and scored the creativity of each idea on a Likert-scale (1: not at all creative–5: highly creative), one after the other. Agreement between raters was good for the first form (Cronbach’s *α* = 0.77) and acceptable for the second form (Cronbach’s *α* = 0.63). We used three dependent variables from this task: fluency, the total number of ideas produced by a participant, the average creativity of ideas on both tasks (creativity ratings of ideas were averaged across raters, summed for each participant, and divided by fluency), and average originality (participants were provided 1 point for each idea that was unique in the sample, and the sum of the points was divided by the number of ideas generated). ‘Just suppose’ task data was missing for 2 participants due to data collection error.

The ability of participants to solve problems using remote associations was probed with the Hungarian version of the Compound Remote Associate problems (CRA; Bowden & Jung-Beeman, 2003; Hungarian adaptation: Simor & Polner, 2017). In each trial of this task, participants are presented three words, and their task is to find a fourth word that can be combined with each of the presented words to create a valid compound word (e.g., AGE/MILE/SAND form the compounds STONEAGE, MILESTONE, and SANDSTONE with the solution word STONE’, (Bowden & Jung-Beeman, 2003 p. 635). The CRA was completed on a laptop using PsychoPy2 (Peirce, 2007). After the instruction, participants completed five practice trials to ensure they understood the task. Then, fifty Hungarian CRA items were presented in random order, one after the other. In the beginning of each trial, a fixation cross was shown, and when participants attended to the screen, the experimenter pressed the spacebar and three words simultaneously appeared above, at, and below the middle of the screen. We instructed participants to say out loud the solution as soon as they had it. If they told the correct response, the experimenter pressed the spacebar, and the next trial began; otherwise the experimenter provided no feedback, and the item stayed on the screen and participants were allowed to try again. Incorrect responses were not recorded. The time limit for solving each item was 30 s, after which the program automatically initiated the next trial. The CRA had good reliability in the present sample (ordinal *α* = 0.73). Data for one participant was missing due to computer error.

#### Schizotypy, general mental health and insomnia

Schizotypal personality traits were assessed with the Hungarian version of the shortened Oxford-Liverpool Inventory of Feelings and Experiences (sO-LIFE Mason, Linney & Claridge, 2005). The sO-LIFE is rooted in the personality approach to schizotypy, and it consists of 43 self-report yes/no items, which belong to four subscales: Unusual Experiences (positive schizotypy: hallucination- and delusion-like experiences; the subscale had excellent internal consistency in the present sample: ordinal *α* = 0.83), Cognitive Disorganisation (disorganised schizotypy: loosened associations and social anxiety; internal consistency was excellent in the present sample: ordinal *α* = 0.88), Introvertive Anhedonia (negative schizotypy: physical anhedonia and social withdrawal; this subscale showed fair internal consistency in the present sample: ordinal *α* = 0.69), and Impulsive Nonconformity (impulsive schizotypy: impulsive, asocial and aggressive tendencies; internal consistency was fair in this sample: ordinal *α* = 0.60).

In order to measure mental health complaints, participants completed the Hungarian version of the General Health Questionnaire (GHQ-12) (Goldberg et al., 1997; Balajti et al., 2007). The GHQ-12 is a widely used scale to screen for mental health problems, such as depressive and anxiety symptoms. The scale is used for representative epidemiological surveys assessing health problems in the Hungarian population, and showed good internal consistency and construct validity in previous studies (Balajti et al., 2007; Simor et al., 2015). The internal consistency of the GHQ-12 was excellent in the present sample (Cronbach’s *α* = 0.88).

Subjective sleep complaints were measured by the Athen Insomnia Scale (AIS; Soldatos, Dikeos & Paparrigopoulos, 2000). The AIS is a reliable and valid instrument for the measurement of subjective sleep quality and complaints of insomnia (Soldatos, Dikeos & Paparrigopoulos, 2000). The first five items of the scale cover night-time symptoms such as difficulties of falling asleep, nocturnal awakenings, perceived sleep duration, early-morning awakening and subjective sleep quality. The last three items refer to the daytime effects of impaired sleep related to bad mood, impaired performance and daytime fatigue. The Hungarian adaptation of the questionnaire (based on the data of 12,000 individuals) proved to be a highly reliable and valid tool in order to assess insomnia symptoms and disrupted sleep in the adult population (Novak, 2004). The scale showed good internal consistency in the present study (Cronbach’s *α* = 0.76).

#### Verbal short term memory and intellect

Verbal short term memory was probed with the forward digit span task (Wechsler, 1981) (Hungarian version: Racsmány et al., 2005). In this task, the experimenter reads aloud sequences of digits, keeping 1 s pause after each digit, and participants are requested to repeat the sequence after the experimenter. The length of the sequences progressively increase; verbal short term memory span is indicated by the maximum sequence length at which a participant is able to perfectly accurately repeat at least 3 out of the 4 different sequences. Digit span data was missing for one participant, which we imputed with the digit span sample mean (6.46).

Intellect is a personality trait closely related to intelligence (DeYoung, Grazioplene & Peterson, 2012; Kaufman, 2013; DeYoung et al., 2014) working memory capacity (DeYoung et al., 2009; Kaufman et al., 2010; Kaufman, 2013) and related neural activity (DeYoung et al., 2009). We measured intellect with the Rational Ability and Engagement subscales of the Rational-Experiential Inventory (REI; Pacini & Epstein, 1999; Hungarian version: Bognár, Orosz & Büki, 2014), which contains 20 Likert items (1: ‘definitely not true of myself’–5: ‘definitely true of myself’) related to reasoning about complex problems, abstract thinking and logical analysis. Internal consistency was excellent for the Ability subscale (Cronbach’s *α* = 0.83) and good for the Engagement subscale (Cronbach’s *α* = 0.78).

### Procedure

Participants applying for the study were invited to the laboratory where they completed the digit span task, the ‘Just suppose’ test and the CRA on an individual basis. Then, they were provided access to the online forms which they filled in the following day.

### Statistical analysis

Data analysis was performed with R (version 3.4.3, R Core Team, 2018) using RStudio (version 1.1.423, RStudio team, 2016). First, before we analysed the relationship between schizotypy, insomnia, mental health, intellect and real-life creative achievements, we excluded 8 participants who reported any current or past neurological or psychiatric disorder, and/or reported intake of medications that were used to treat mental disorders (e.g., selective serotonin reuptake inhibitors), and 2 participants who reported past/current dyslexia. Furthermore, we excluded 4 participants who were older than 30 years (age range: 36–50) in order to make our sample more homogeneous with respect to age. Thus, we analysed the relationships between questionnaire data on a sample of 168 participants (mean (sd) of age = 21.6 (2.4); 58% female). Second, when the models involved performance laboratory-based tasks, we applied additional exclusion criteria. For these analyses, we excluded participants who reported that 24 hours prior to testing have (a) slept less than 6 hours in the night (in order to minimize confounding by current sleep deprivation); *N* = 16, (b) taken medication that is likely to affect alertness and cognition (such as painkillers and drugs used to treat allergies and hypertension); *N* = 9, or (c) taken an excessive amount of caffeine (4 or more units); *N* = 2. These criteria permitted the analysis of performance on laboratory-based tasks in a sample of 141 participants (mean (sd) of age = 21.6 (2.3); 59% female, further descriptive statistics are shown in Table 1).

**Table 1:** Descriptive statistics.

Variable	Mean	Median	Min	Max	sd	Skewness	Kurtosis
Unusual experiences	5.07	5	0	12	2.61	0.27	−0.37
Cognitive disorganisation	5.54	5	0	11	2.91	0	−0.9
Introvertive anhedonia	1.51	1	0	7	1.55	1.16	1.04
Impulsive nonconformity	4.45	4	0	9	1.8	0.3	−0.21
Creative achievements	8.11	6	0	42	7.39	1.7	3.51
Creative achievements in art	6.06	4	0	36	6.68	2.06	4.9
Creative achievements in science	1.3	0	0	20	2.76	3.51	15.96
Remote association problem solving	20.44	20	7	33	5.26	0.08	−0.24
Divergent thinking (average creativity)	4.56	4.33	3.11	9.44	0.98	1.6	4.21
Divergent thinking (average originality)	0.48	0.45	0	2	0.42	1.18	1.83
Divergent thinking (fluency)	10.24	10	2	30	4.4	0.98	1.99
Mental health	35.03	36	12	47	6.84	−0.86	0.62
Insomnia	5.08	5	0	20	3.37	1.18	2.53
Rational ability	36.91	37	19	49	6.3	−0.23	−0.49
Rational engagement	38.63	39	19	48	5.58	−0.84	1.09
Digit span	6.46	7	4	9	1.08	−0.03	−0.53

A Spearman-correlation matrix was calculated to explore zero-order associations between schizotypy, general mental health, insomnia, digit span, intellect and creative achievements and creative thinking (using pairwise complete observations). Then, we performed a series of multiple regressions to predict the following creativity-related variables: (1) real-life creative achievements (in total, in art, and in science), (2) remote associative problem solving, (3) fluency, (4) creativity and (5) originality of divergent thinking. Positive, disorganised, impulsive and negative schizotypy scores were entered in Block 1, along with age, gender and smoking status. In Block 2, general mental health, insomnia, digit span and intellect were added as additional predictors. Variance inflation factors (VIF) were calculated to examine multicollinearity; VIF > 5 indicates problematic multicollinearity (James et al., 2013). As creative achievement, negative schizotypy and divergent thinking scores were strongly skewed, we log10-transformed these variables for the regression analyses.

## Results

The zero-order Spearman-correlation matrix of variables reflecting schizotypy, general mental health, insomnia, digit span, intellect and creative achievement and creative thinking skills is shown in Table 2. Dimensions of schizotypy were negatively related to mental health and positively to insomnia. Disorganised and negative schizotypy were negatively related to measures of intellect, which, in turn, were positively related not only to mental health and adequate sleep, but also to creative accomplishments and creativity of ideas on the divergent thinking task. Mental health was moderately and positively associated with creative achievements, and insomnia showed a moderate and negative correlation with remote association problem solving, although these correlations did not reach statistical significance.

**Table 2:** Correlations between dimensions of schizotypy, mental health, insomnia, digit span, intellect and indicators of creativity.

	2	3	4	5	6	7	8	9	10	11	12	13	14	15	16
1. Unusual experiences	0.39^***^	−0.05	0.36^***^	−0.35^***^	0.33^***^	−0.04	−0.1	0.1	0.09	0.13	0.02	−0.09	0.09	0.08	−0.13
2. Cognitive disorganisation		0.37^***^	0.35^***^	−0.52^***^	0.5^***^	−0.05	−0.37^***^	−0.18^*^	−0.02	−0.01	−0.01	0.13	0.07	−0.07	0.03
3. Introvertive anhedonia			0.01	−0.26^***^	0.24^**^	0.03	−0.18^*^	−0.28^***^	−0.07	−0.06	−0.06	0.01	<0.01	−0.05	0.11
4. Impulsive nonconformity				−0.22^**^	0.26^***^	0.01	−0.07	0.01	0.02	−0.01	0.05	0.12	−0.05	−0.08	−0.01
5. Mental health					−0.58^***^	0.04	0.3^***^	0.13	0.14	0.07	0.17^*^	0.1	−0.11	0.04	0.01
6. Insomnia						−0.06	−0.26^***^	−0.18^*^	−0.06	−0.03	−0.1	−0.14	−0.01	−0.03	−0.08
7. Digit span							0.11	0.07	0.15	0.09	0.12	0.21^*^	0.13	0.06	0.1
8. Rational ability								0.55^***^	0.29^***^	0.2^*^	0.33^***^	0.01	0.23^**^	0.02	0.14
9. Rational engagement									0.2^**^	0.15	0.21^**^	0.07	0.35^***^	−0.12	0.16
10. Creative achievements										0.88^***^	0.34^***^	0.1	0.22^**^	0.05	0.19^*^
11. Creative achievements (art)											<0.01	0.07	0.19^*^	0.06	0.18^*^
12. Creative achievements (science)												−0.02	0.16	−0.07	0.05
13. Remote associations													0.11	0.02	0.09
14. Divergent t. (creativity)														−0.13	0.63^***^
15. Divergent t. (fluency)															−0.02
16. Divergent t. (originality)															

**Notes.**

Spearman-rho correlation coefficients are shown, computed using pairwise complete observations.

*** *p* < 0.001

** *p* < 0.01

* *p* < 0.05

### Creative achievements

In the first block, real-life creative achievements were positively and significantly predicted by positive schizotypy (Unusual Experiences), and negatively by smoking (Table 3). In the second block, intellect (Rational Ability) also yielded a significant positive effect, while the effects of schizotypy dimensions were essentially unchanged. Both models showed significant fit, and the improvement of fit in the second block was significant. Creative achievements in art were positively predicted by positive schizotypy (Unusual Experiences) and intellect (Rational Ability), and negatively by smoking (Table 4). Furthermore, although creativity in science and inventions was not significantly associated with schizotypy in the first block, disorganised schizotypy (Cognitive Disorganisation) emerged as a positive predictor in the second block, while intellect (Rational Ability) also yielded a significant positive effect (Table 5). No sign of problematic multicollinearity was observed in any of the models.

**Table 3:** Predicting real-life creative achievements.

Predictor	Beta	SE	*t*	*p*	VIF	Model statistics
*Block 1*						
(Intercept)	0.619	0.313	1.98	0.05		*R*^2^=0.09, *R*^2^*adj*=0.05
**Unusual Experiences**	**0.028**	**0.013**	**2.25**	**0.026**	**1.3**	*F* (8,133) =1.96, *p*=0.065
Cognitive disorganisation	−0.004	0.013	−0.33	0.739	1.6	
Introvertive anhedonia	−0.077	0.127	−0.61	0.543	1.3	
Impulsive nonconformity	0.004	0.018	0.21	0.833	1.3	
**Smoking**	**−0.249**	**0.084**	**−2.94**	**0.004**	**1.1**	
Gender (female)	−0.046	0.066	−0.7	0.485	1.3	
Age	0.008	0.013	0.57	0.566	1.1	
*Block 2*						
(Intercept)	−0.612	0.46	−1.33	0.186		*R*^2^=0.19, *R*^2^*adj*=0.12
**Unusual experiences**	**0.029**	**0.013**	**2.3**	**0.023**	**1.4**	*F* (13,128) =2.53, *p* = 0.005
Cognitive disorganisation	0.011	0.014	0.84	0.403	2	*R*^2^ change =0.1, *F* (5,128) =3.1, *p* = 0.011
Introvertive anhedonia	−0.059	0.126	−0.47	0.639	1.4	
Impulsive nonconformity	0.003	0.018	0.17	0.867	1.3	
**Smoking**	**−0.262**	**0.085**	**−3.08**	**0.002**	**1.2**	
Gender (female)	0.002	0.066	0.02	0.981	1.4	
Age	0.008	0.013	0.64	0.523	1.1	
Mental health	0.005	0.005	0.89	0.374	1.6	
Insomnia	0.002	0.01	0.2	0.841	1.6	
**Rational ability**	**0.015**	**0.006**	**2.54**	**0.012**	**1.7**	
Rational engagement	0.002	0.006	0.3	0.761	1.6	
Digit span	0.049	0.026	1.87	0.063	1	

**Table 4:** Predicting real-life creative achievements in art.

Predictor	Beta	SE	*t*	*p*	VIF	Model statistics
*Block 1*						
(Intercept)	0.488	0.337	1.45	0.15		*R*^2^=0.09, *R*^2^*adj*=0.05
**Unusual experiences**	**0.036**	**0.014**	**2.68**	**0.008**	**1.3**	*F* (8,133) =1.97, *p* = 0.064
Cognitive disorganisation	−0.011	0.014	−0.82	0.417	1.6	
Introvertive anhedonia	0.041	0.136	0.3	0.762	1.3	
Impulsive nonconformity	−0.008	0.02	−0.4	0.692	1.3	
**Smoking**	**−0.212**	**0.091**	**−2.33**	**0.021**	**1.1**	
Gender (female)	0.05	0.071	0.7	0.485	1.3	
Age	0.005	0.014	0.34	0.734	1.1	
*Block 2*						
(Intercept)	−0.528	0.505	−1.05	0.298		*R*^2^=0.16, *R*^2^*adj*=0.08
**Unusual experiences**	**0.04**	**0.014**	**2.83**	**0.005**	**1.4**	*F* (13,128) =2.01, *p*=0.029
Cognitive disorganisation	0.002	0.015	0.15	0.88	2	*R*^2^ change =0.06, *F* (5,128) =1.96, *p* = 0.089
Introvertive anhedonia	0.043	0.138	0.31	0.755	1.4	
Impulsive nonconformity	−0.01	0.019	−0.49	0.622	1.3	
**Smoking**	**−0.219**	**0.093**	**−2.34**	**0.021**	**1.2**	
Gender (female)	0.096	0.073	1.32	0.189	1.4	
Age	0.007	0.014	0.47	0.641	1.1	
Mental health	0.004	0.006	0.72	0.47	1.6	
Insomnia	0	0.011	0.02	0.98	1.6	
**Rational ability**	**0.013**	**0.006**	**2.13**	**0.035**	**1.7**	
Rational engagement	−0.002	0.007	−0.37	0.715	1.6	
Digit span	0.05	0.029	1.75	0.082	1	

**Table 5:** Predicting real-life creative achievements in science.

Predictor	Beta	SE	*t*	*p*	VIF	Model statistics
*Block 1*						
(Intercept)	0.431	0.269	1.6	0.112		*R*^2^=0.17, *R*^2^*adj*=0.13
Unusual experiences	−0.003	0.011	−0.26	0.792	1.3	*F* (8,133) =3.92, *p* = 0.001
Cognitive disorganisation	0.018	0.011	1.68	0.095	1.6	
Introvertive anhedonia	−0.207	0.109	−1.9	0.06	1.3	
Impulsive nonconformity	0.004	0.016	0.28	0.778	1.3	
**Smoking**	**−0.188**	**0.073**	**−2.6**	**0.01**	**1.1**	
**Gender (female)**	**−0.269**	**0.057**	**−4.72**	**<0.001**	**1.3**	
Age	−0.003	0.011	−0.3	0.768	1.1	
*Block 2*						
(Intercept)	−0.466	0.398	−1.17	0.244		*R*^2^=0.25, *R*^2^*adj*=0.18
Unusual experiences	−0.003	0.011	−0.31	0.759	1.4	*F* (13,128) =3.54, *p* < 0.001
**Cognitive disorganisation**	**0.034**	**0.012**	**2.87**	**0.005**	**2**	*R*^2^ change =0.08, *F* (5,128) =2.67, *p* = 0.025
Introvertive anhedonia	−0.174	0.109	−1.59	0.113	1.4	
Impulsive nonconformity	0.005	0.015	0.3	0.761	1.3	
**Smoking**	**−0.186**	**0.074**	**−2.53**	**0.013**	**1.2**	
**Gender (female)**	**−0.235**	**0.058**	**−4.09**	**<0.001**	**1.4**	
Age	−0.004	0.011	−0.32	0.75	1.1	
Mental health	0.003	0.004	0.63	0.531	1.6	
Insomnia	−0.005	0.009	−0.5	0.614	1.6	
**Rational ability**	**0.011**	**0.005**	**2.28**	**0.024**	**1.7**	
Rational engagement	0.005	0.005	0.88	0.378	1.6	
Digit span	0.017	0.023	0.75	0.452	1	

### Problem solving using remote associations

In the first block, the model fit was non-significant. Entering variables in the second block caused a significant improvement of model fit (see Table 6). Disorganised schizotypy (Cognitive Disorganisation) and digit span positively predicted performance on the CRA, while insomnia (AIS) had a negative effect. No problematic multicollinearity was indicated by VIF values.

**Table 6:** Predicting performance on the Compound Remote Associates task.

Predictor	Beta	SE	*t*	*p*	VIF	Model statistics
*Block 1*						
(Intercept)	11.905	4.739	2.51	0.013		*R*^2^=0.09, *R*^2^*adj*=0.04
**Unusual experiences**	**−0.444**	**0.191**	**−2.32**	**0.022**	**1.3**	*F* (8,132) =1.79, *p* = 0.095
Cognitive disorganisation	0.273	0.191	1.43	0.155	1.6	
Introvertive anhedonia	−1.274	1.919	−0.66	0.508	1.3	
Impulsive nonconformity	0.345	0.276	1.25	0.213	1.3	
Smoking	1.502	1.277	1.18	0.242	1.1	
Gender (female)	0.997	1.003	0.99	0.322	1.3	
Age	0.339	0.199	1.71	0.09	1.1	
*Block 2*						
(Intercept)	1.491	6.951	0.21	0.831		*R*^2^=0.2, *R*^2^*adj*=0.12
Unusual experiences	−0.323	0.192	−1.68	0.095	1.4	*F* (13,127) =2.61, *p* = 0.004
**Cognitive disorganisation**	**0.537**	**0.204**	**2.64**	**0.009**	**2**	*R*^2^ change =0.11, *F* (5,127) =3.53, *p* = 0.005
Introvertive anhedonia	−0.535	1.894	−0.28	0.778	1.4	
Impulsive nonconformity	0.324	0.265	1.22	0.223	1.3	
Smoking	2.289	1.274	1.8	0.075	1.2	
Gender (female)	1.052	0.998	1.05	0.294	1.4	
Age	0.337	0.192	1.75	0.082	1.1	
Mental health	0.073	0.077	0.95	0.346	1.6	
**Insomnia**	**−0.405**	**0.157**	**−2.58**	**0.011**	**1.6**	
Rational ability	−0.015	0.086	−0.18	0.86	1.7	
Rational engagement	0.053	0.094	0.56	0.574	1.5	
**Digit span**	**0.949**	**0.395**	**2.4**	**0.018**	**1**	

**Table 7:** Predicting average creativity of ideas on the ‘Just suppose’ task.

Predictor	Beta	SE	*t*	*p*	VIF	Model statistics
*Block 1*						
(Intercept)	0.638	0.065	9.77	<0.001		*R*^2^=0.03, *R*^2^*adj*= −0.02
Unusual experiences	0.003	0.003	0.96	0.342	1.3	*F* (8,131) =0.65, *p* = 0.71
Cognitive disorganisation	0.001	0.003	0.48	0.631	1.6	
Introvertive anhedonia	0.01	0.026	0.36	0.715	1.3	
Impulsive nonconformity	−0.001	0.004	−0.36	0.721	1.3	
Smoking	−0.013	0.018	−0.75	0.455	1.1	
Gender (female)	−0.001	0.014	−0.08	0.932	1.3	
Age	0.004	0.003	1.47	0.145	1.1	
*Block 2*						
(Intercept)	0.443	0.094	4.73	<0.001		*R*^2^=0.18, *R*^2^*adj*=0.1
Unusual experiences	<0.001	0.003	0.15	0.881	1.5	*F* (13,126) =2.27, *p* = 0.012
Cognitive disorganisation	0.004	0.003	1.49	0.138	2	*R*^2^ change =0.14, *F* (5,126) =4.41, *p* = 0.001
Introvertive anhedonia	0.014	0.026	0.55	0.585	1.4	
Impulsive nonconformity	<0.001	0.004	−0.02	0.983	1.3	
Smoking	−0.021	0.017	−1.19	0.235	1.3	
Gender (female)	0.004	0.014	0.31	0.758	1.4	
Age	0.003	0.003	1.27	0.207	1.1	
Mental health	−0.001	0.001	−1.16	0.247	1.7	
Insomnia	−0.002	0.002	−0.92	0.359	1.6	
Rational ability	0.002	0.001	1.43	0.155	1.7	
**Rational engagement**	**0.004**	**0.001**	**2.85**	**0.005**	**1.5**	
Digit span	0.008	0.005	1.43	0.155	1	

**Table 8:** Predicting average originality of ideas on the ‘Just suppose’ task.

Predictor	Beta	SE	*t*	*p*	VIF	Model statistics
*Block 1*						
(Intercept)	0.077	0.109	0.71	0.481		*R*^2^=0.03, *R*^2^*adj*= −0.03
Unusual experiences	−0.004	0.004	−0.89	0.375	1.3	*F* (8,131) = 0.5, *p* = 0.83
Cognitive disorganisation	<0.001	0.004	−0.08	0.941	1.6	
Introvertive anhedonia	0.037	0.044	0.85	0.395	1.3	
Impulsive nonconformity	0.005	0.006	0.76	0.448	1.3	
Smoking	−0.019	0.029	−0.65	0.518	1.1	
Gender (female)	0.018	0.023	0.77	0.444	1.3	
Age	0.003	0.005	0.61	0.543	1.1	
*Block 2*						
(Intercept)	−0.149	0.163	−0.92	0.36		*R*^2^=0.09, *R*^2^*adj*=0.01
Unusual experiences	−0.006	0.005	−1.27	0.206	1.5	*F* (13,126) =1.09, *p*=0.374
Cognitive disorganisation	0.003	0.005	0.72	0.47	2	*R*^2^ change =0.07, *F* (5,126) =1.89, *p* = 0.101
Introvertive anhedonia	0.044	0.045	0.97	0.332	1.4	
Impulsive nonconformity	0.006	0.006	0.98	0.33	1.3	
Smoking	−0.024	0.03	−0.79	0.43	1.3	
Gender (female)	0.023	0.023	0.99	0.323	1.4	
Age	0.002	0.005	0.45	0.655	1.1	
Mental health	−0.001	0.002	−0.62	0.536	1.7	
Insomnia	−0.004	0.004	−0.97	0.336	1.6	
Rational ability	0.002	0.002	0.81	0.421	1.7	
Rational engagement	0.004	0.002	1.76	0.081	1.5	
Digit span	0.011	0.009	1.17	0.246	1	

**Table 9:** Predicting fluency on the ‘Just suppose’ task.

Predictor	Beta	SE	*t*	*p*	VIF	Model statistics
*Block 1*						
(Intercept)	1.167	0.163	7.18	<0.001		*R*^2^=0.02, *R*^2^*adj*= −0.03
Unusual experiences	0.007	0.007	1	0.317	1.3	*F* (8,131) =0.38, *p* = 0.91
Cognitive disorganisation	−0.005	0.007	−0.72	0.473	1.6	
Introvertive anhedonia	0.009	0.066	0.13	0.895	1.3	
Impulsive nonconformity	−0.002	0.009	−0.26	0.797	1.3	
Smoking	−0.027	0.044	−0.61	0.541	1.1	
Gender (female)	−0.018	0.034	−0.52	0.604	1.3	
Age	−0.006	0.007	−0.91	0.366	1.1	
*Block 2*						
(Intercept)	1.17	0.248	4.72	<0.001		*R*^2^=0.06, *R*^2^*adj*= −0.03
Unusual experiences	0.01	0.007	1.47	0.143	1.5	*F* (13,126) =0.62, *p* = 0.819
Cognitive disorganisation	−0.005	0.007	−0.7	0.488	2	*R*^2^ change =0.04, *F* (5,126) =0.96, *p* = 0.445
Introvertive anhedonia	−0.01	0.068	−0.14	0.889	1.4	
Impulsive nonconformity	−0.004	0.01	−0.44	0.662	1.3	
Smoking	−0.023	0.046	−0.49	0.625	1.3	
Gender (female)	−0.009	0.036	−0.25	0.804	1.4	
Age	−0.004	0.007	−0.63	0.528	1.1	
Mental health	0.001	0.003	0.51	0.612	1.7	
Insomnia	0.001	0.006	0.19	0.848	1.6	
Rational ability	0.002	0.003	0.62	0.54	1.7	
Rational engagement	−0.006	0.003	−1.93	0.056	1.5	
Digit span	0.011	0.014	0.76	0.448	1	

### Divergent thinking

None of the divergent thinking indicators (creativity, originality, or fluency) was significantly predicted by any dimension of schizotypy in any of the models (see Tables 7–9). Only the model predicting rated creativity of ideas on the divergent thinking task turned significant in the second, due to the significant positive effect of intellect (Rational Engagement) (Table 7).

## Discussion

In this study, we tested the hypothesis that the association between positive, disorganised and impulsive schizotypy and indicators related to creativity will become more apparent if adverse correlates of schizotypy, such as impaired general mental health, insomnia, and reduced intellect, are statistically controlled. Our findings provided partial support for this hypothesis and yielded a somewhat surprising finding. First, we have found that positive schizotypy significantly and positively predicted real-life creative achievements. Second, contrary to the findings of a recent meta-analysis (Acar & Sen, 2013), we have found that disorganised schizotypy significantly and positively predicted creative achievements in science (when the positive effect of intellect was controlled for) and remote association problem solving performance (if the negative effect of insomnia was partialled out). In line with the meta-analysis of Acar & Sen (2013), the observed effects were small, but significant. Third, no dimension of schizotypy was significantly associated with fluency, creativity or originality of divergent thinking, not even when the effects of general mental health, insomnia, verbal short term memory or intellect were statistically controlled.

Previous studies have demonstrated that positive schizotypy is associated with unusual experiences that facilitate the creative process. Nelson & Rawlings (2010) found elevated levels of positive schizotypy in a sample of 100 hundred artists, and positive schizotypy was a robust predictor of immersion in creating art and the associated sense of anxiety, and also of power and pleasure experienced during performing creative activities. A recent experience sampling study also found elevated positive schizotypy among artists (Holt, in press), and in this sample, positive schizotypy predicted experiences of flow, vivid visual imagery, introspection, and absorption in daily life. Importantly, positive schizotypy was not only correlated with art-making and inspiration, but it was also associated with increased positive affect and self-esteem during and after creating artwork. Aberrant salience (i.e., attribution of meaning and significance to neutral stimuli) could be a common neurocognitive mechanism that may promote unusual experiences and ideas during the creative process and could also play a role in psychotic-like experiences associated with positive schizotypy (Nelson et al., 2014).

The positive association between disorganised schizotypy with remote associate problem solving and creative achievements in science might seem unexpected: a meta-analysis reported a weak and significant negative association between negative-disorganised schizotypy and creativity (Acar & Sen, 2013). However, our findings are in line with the relevance of schizotypal features such as overinclusive thinking (Eysenck, 1993) and flat associative hierarchy (Mednick, 1962) to creativity. Enhanced semantic priming (Mednick, Mednick & Mednick, 1964; Bowden et al., 2005) and high levels of intelligence and cognitive control (Chein & Weisberg, 2014; Lee, Huggins & Therriault, 2014) are both associated with success at solving remote association problems. Importantly, enhanced semantic priming has been documented in patients with thought disorder, a condition representing the most severe end of disorganisation in the psychosis-spectrum (Spitzer, 1997; Moritz et al., 2003). Likewise, disorganised schizotypy (odd behaviour and speech) has been positively related to higher rate of associational connections in speech (Lenzenweger et al., 2007), while cognitive disorganisation has been associated with enhanced indirect semantic priming (Johnston, Rossell & Gleeson, 2008) and more errors on a semantic categorisation task (Morgan et al., 2009). Thus, our data implies that semantic hyperpriming could promote remote associate problem solving if the negative effects of accompanying sleep disturbances or reduced intellect (likely to be mediated by impaired cognitive functions) are controlled. The controversy between the present and the meta-analytic findings (Acar & Sen, 2013) with respect to disorganised schizotypy might be resolved in multiple ways: (1) disorganisation measured by the O-LIFE is conceptually and empirically different from disorganisation measured by the SPQ—the former measures cognitive slippage resembling formal thought disorder, and social anxiety, while the latter contains items concerned with eccentric behaviour and speech (Oezgen & Grant, 2018); (2) Acar & Sen (2013) merged disorganisation and negative schizotypy in their meta-analysis, and negative schizotypy has been shown to be associated with impaired updating and shifting by a recent a meta-analysis (Steffens et al., 2018); (3) the meta-analysis by Acar & Sen (2013) did not consider the potential confounding effects of general psychopathology and insomnia. Further studies should attempt to replicate whether controlling for insomnia and intellect would yield a positive association between disorganisation, and remote association problem solving and creative achievements in science.

We found no significant association between verbal divergent thinking and schizotypy, not even when we controlled for adverse correlates of schizotypy. The lack of significant findings could be due to insufficient statistical power to detect a weak effect. A meta-analysis implied a weak relationship between schizotypy and creativity (Acar & Sen, 2013), and divergent thinking might be less reliant on mental health and cognitive control, relative to creative achievements and remote association problem solving (e.g., Lee, Huggins & Therriault, 2014). Alternatively, the null findings might be explained by the limited reliability of the divergent thinking task we applied: Silvia (2011) have found that the scores on the unusual uses task were more reliable than scores on the consequences task (a task conceptually similar to the ‘Just suppose’ task). However, good-acceptable inter-rater agreement and the significant positive correlation between real-life creative achievements and average creativity of divergent thinking support the reliability and the validity of the average creativity score in our sample. It is also important to note that exploratory analyses conducted by Acar & Sen (2013) suggested that schizotypy may be particularly associated with artistic creativity and the effects might be larger in samples of outstanding creative achievers. Finally, one may speculate that the instruction we gave prior to the divergent thinking task might have reduced the association between schizotypy and creativity/originality of ideas. The ‘Just suppose’ was framed as a game-like task, and the instruction simultaneously emphasised the quality and the quantity of ideas: participants were asked to produce as many novel ideas as they could, and were also encouraged to ‘try to come up with ideas that nobody else would have’. The “be-creative” instruction and the relaxed atmosphere may not only increase the creativity of responses, but can also enhance criterion validity of the scores (e.g., Vernon, 1971; Harrington, 1975; Katz & Poag, 1979; Nusbaum, Silvia & Beaty, 2014). Asocial-impulsive schizotypy (and psychoticism) have been suggested to be related to the propensity to express shocking, unconventional ideas, while positive schizotypy might be related to having unusual ideas (Rawlings & Toogood, 1997; Burch et al., 2006; Acar & Runco, 2012). Thus, the associations between positive and impulsive-asocial schizotypy and divergent thinking might be more likely to emerge if participants are not explicitly instructed to produce original and creative ideas; such instructions might trigger low schizotypy participants to think in unusual ways and to express unconventional ideas.

## Conclusions

A particular strength of this study is that it applied a multidimensional and nuanced approach to investigate the relationship between schizotypy and creativity. Taking into account mental health impairment, insomnia and intellectual capacities have revealed a specific, previously unknown pattern of associations between disorganised schizotypy and indicators of creativity. A limitation of the study is that schizotypy, mental health and insomnia were assessed with self-report scales, which are more prone to bias, relative to clinical interviews (Linscott & Van Os, 2010). Furthermore, we only measured subjective sleep quality and there were no objective assessments of sleep patterns. Nevertheless, subjective sleep quality as measured by simple questionnaires is a quite sensitive index of disrupted sleep and predictive of a variety of adverse health outcomes (Riemann et al., 2017; Short, Allan & Schmidt, 2017). Further research, however, should corroborate and extend these findings by including the measurement of objective sleep parameters. Moreover, future studies should characterise the dynamics of causality between sleep disturbances and schizotypy (Barton et al., 2018) and also between creative accomplishments and mental health (Amabile et al., 2005; Holt, in press).

##  Supplemental Information

10.7717/peerj.5615/supp-1 Supplemental Information 1 Raw data for 182 participants

10.7717/peerj.5615/supp-2 Supplemental Information 2 R code for data analysis

10.7717/peerj.5615/supp-3 Supplemental Information 3 Codebook Contains variable labels

## Additional information and Declarations

### Data Availability

The following information was supplied regarding data availability: The raw data and code are provided in the Supplemental Files.
